# Impact of variety and environmental factors on proteolytic quality parameters of selected malting barley varieties

**DOI:** 10.1002/jsfa.70866

**Published:** 2026-07-07

**Authors:** Shari Piro, Martina Gastl, Michael Féchir

**Affiliations:** ^1^ Trier University of Applied Science Trier Germany; ^2^ Technical University of Munich TUM Research Center Weihenstephan for Brewing and Food Quality Freising‐Weihenstephan Germany; ^3^ Bitburger Brewery Group Bitburg Germany; ^4^ KU Leuven Ghent Belgium

**Keywords:** environmental impact, industrial dataset, malt quality, malting barley, proteolytic malt characteristics

## Abstract

**BACKGROUND:**

Barley (*Hordeum vulgare*) is a major cereal crop, essential for global food processing and brewing. Malting barley represents less than one‐fifth of global barley output due to strict quality requirements, such as total protein content, enzymatic activity, and kernel homogeneity, which directly influence malting performance and, consequently, beer quality. Despite enormous progress in breeding for agronomic performance and grain quality, increasing climate variability poses significant challenges to malting barley production, affecting both yield and quality. An understanding of the ways in which environmental and genotypic factors influence protein composition is critical to ensure consistent malt quality. This study analyzed malt quality data from 7458 malt batches representing six commercial malting barley varieties harvested between 2017 and 2024 across three European regions to quantify, evaluate, and contextualize the effects of environmental and genotypic factors on key proteolytic quality parameters, including total protein content, soluble nitrogen (SN), free amino nitrogen (FAN), and the Kolbach index (KI).

**RESULTS:**

Proteolytic parameters (PPs) followed a sequential cascade from total protein to SN and FAN. Total protein varied mainly among harvest year × variety combinations, whereas SN and FAN showed pronounced temporal declines, particularly during 2021–2024. Varieties 1 and 5 combined high SN with high FAN, whereas variety 3 consistently showed the lowest values. Variety 6 displayed the highest FAN despite intermediate SN, indicating genotype‐specific proteolytic efficiency. The KI showed low variability and confirmed classification into high‐ and moderate modification groups. Regional effects were minor.

**CONCLUSION:**

The decline in SN and FAN in more recent years, despite variable protein levels, indicates that protein quality is a more important determinant of malt performance than total protein quantity alone. This cascade‐oriented perspective provides a clearer interpretation of quality variation in malting barley and supports more informed decisions regarding variety selection and raw‐material management. © 2026 The Author(s). *Journal of the Science of Food and Agriculture* published by John Wiley & Sons Ltd on behalf of Society of Chemical Industry.

**ABBREVIATIONS**
BLQ: Research Center Weihenstephan for Brewing and Food Quality
FAN: free amino nitrogen
IPCC: Intergovernmental Panel on Climate Change
KI: Kolbach index
PCA: principal component analysis
PPs: proteolytic parameters
SN: soluble nitrogen
SN‐65: soluble nitrogen in 65 °C mash
SN‐C: soluble nitrogen in congress mash

## INTRODUCTION

Barley (*Hordeum vulgare*) is one of the world's most important cereal crops and is widely used in food processing and brewing. Global production reached approximately 143.3 million t in the 2024/2025 season.^1^ From 1994 to 2023, Russia was the world's leading barley producer, followed by Germany and France, highlighting the strategic roles of Germany and France in shaping both regional and global markets.^2^ In contrast to feed and food barley, barley intended for malting and brewing must meet particularly stringent quality requirements to ensure processability.^3^ Consequently, only a small proportion of total barley production is suitable for this purpose, with malting barley accounting for less than one‐fifth of global barley output.^4^ The quality of malting barley is crucial for brewers because it directly affects malting and brewing performance, processability, and the characteristics of the final product.^5^, ^6^ High‐quality barley provides the biochemical and structural basis for an efficient malting. Agronomic and compositional characteristics, particularly protein content, kernel uniformity, and starch concentration, strongly influence water uptake during malting, germination kinetics, and the potential for uniform modification.^5^, ^6^, ^7^, ^8^ When barley quality is suboptimal, extract potential may be reduced and endosperm degradation impaired, which limits the degree of modification that can be achieved during germination.^7^, ^8^

During the conversion of barley into malt, proteolytic parameters (PPs), including total protein, soluble nitrogen (SN), free amino nitrogen (FAN), and the Kolbach index (KI) become critical determinants of starch accessibility to enzymes and protein modification during mashing. These attributes determine the suitability of malt for consistent wort production.^5^, ^9^, ^10^, ^11^ Insufficient raw material quality or incomplete modification can therefore reduce enzymatic potential and increase variability in wort composition.^8^, ^9^, ^10^ These deficits can subsequently affect the brewing process. Poorly modified malt may impair lautering and filtration, reduce extract yield, and promote the formation of undesirable flavor‐active or haze‐forming compounds during fermentation and maturation.^10^, ^12^, ^13^, ^14^, ^15^ These effects compromise processing efficiency, which is a critical factor for large‐scale breweries, as well as beer quality and stability.^12^, ^13^, ^16^, ^17^

Malting barley quality is strongly influenced by complex interactions between environmental and genetic factors. In this study, environmental factors are considered in an integrative manner and encompass site‐specific conditions associated with the geographical region of origin and harvest year rather than individual climatic variables. Environmental variability among regions and harvest years represents a major source of variation in barley yield and quality.^18^, ^19^, ^20^, ^21^, ^22^ Differences in growing conditions, particularly during critical developmental stages such as grain filling, can influence key biochemical processes, including protein synthesis, nitrogen assimilation, and storage compound accumulation.^20^, ^23^, ^24^, ^25^, ^26^ These processes ultimately affect the modification potential of barley during malting and the balance between soluble and insoluble protein fractions.^7^, ^8^, ^9^, ^10^ In addition to these environmental influences, increasingly strict agricultural policies, particularly the German Fertilizer Ordinance with its additional restrictions in designated ‘red zones’ and the nutrient‐reduction targets of the EU Green Deal 2030, are expected to further restrict nitrogen inputs. This trend is likely to reduce protein levels in malting barley and reshape quality expectations throughout the supply chain.^27^, ^28^

Genetic factors, reflected in varietal differences, also contribute substantially to variation in barley protein composition. Environmental factors interact with genotype‐specific characteristics, including composition, enzymatic profiles, and stress tolerance.^7^, ^18^, ^23^, ^29^, ^30^, ^31^, ^32^, ^33^, ^34^ In multifactorial datasets, genotype‐specific effects consistently account for a substantial proportion of the variation in barley total protein and other key proteolytic quality parameters of malting barley. These genetic effects often interact with year‐ and region‐specific conditions to shape malting‐relevant protein characteristics.^7^, ^18^, ^19^, ^20^, ^21^, ^22^, ^35^ Understanding how environmental and genetic factors jointly shape protein formation is therefore essential for predicting functional quality and optimizing breeding and sourcing strategies. It also underscores the importance of understanding how specific growing conditions interact with barley genotype to influence protein composition and the functionality of both barley and the resulting malt.^7^, ^18^, ^33^, ^34^

The aim of this study was to systematically quantify, evaluate, and contextualize the relative contributions of environmental and varietal factors to key proteolytic quality parameters in malting barley. Using a comprehensive dataset comprising more than 7000 industrial malt batches, this analysis evaluated the effects of environmental and varietal factors on key proteolytic parameters and examined their interactions. The results of this study are expected to provide practical insights for barley breeding, raw material sourcing, and quality management. They also support decision‐making to help ensure consistent malt quality under increasing climate variability and environmental stress.

## MATERIALS AND METHODS

### Data source

Data from 7458 industrial malt batches representing six commercial barley varieties harvested between 2017 and 2024 and sourced from three European regions (France, Germany, and Scandinavia) were included in the study. The barley was processed into malt in accordance with guaranteed malting barley quality specifications, with target values for friability and SN, adjusted as appropriate for each batch.^36^ All subsequent analyses were performed on the resulting malt. Each malt batch was characterized by PPs, including total protein content, SN in congress mash (SN‐C), SN in 65 °C mash (SN‐65), FAN, and the KI (calculated value). Laboratory analyses were conducted at Avangard Malz AG, Germany, across three production plants in accordance with the respective Mitteleuropäische Brautechnische Analysenkommission (MEBAK) protocols. The total protein content in the malt was determined according to method R‐200.20.030, analogous to method R‐110.41.030 in barley.^37^, ^38^ Wort was prepared using methods R‐205.00.004 and R‐206.00.002 for the congress mash and R‐207.00.002 for the 65 °C mash.^39^, ^40^, ^41^ Measurements of SN‐C and SN‐65 were conducted in accordance with method R‐205.11.030.^42^ The KI value was calculated following guideline R‐205.12.999, while FAN was determined according to method R‐205.14.111.^43^, ^44^

### Data preparation

Raw data were examined for completeness and plausibility. Values outside biologically reasonable limits or flagged by the laboratory were excluded. Categorical factors such as barley variety, harvest year, and region of origin were treated as fixed effects. Only factor combinations with a sample size of ten or more were retained to ensure statistical relevance. Unless stated otherwise, no additional transformations were applied beyond the standardization procedures described below.

### Statistical analysis

Descriptive statistics were calculated for all PPs, including means and standard deviations. Boxplots were generated using GraphPad Prism 5 (GraphPad Software, San Diego, CA) to visualize data distributions and identify potential outliers. The effects of the categorical factors harvest year, barley variety, and region of origin on PPs, together with their interactions up to the third order, were assessed using partial eta‐squared (*η*p^2^) analysis of variance (ANOVA) in XLSTAT (version 2024.4.1, Addinsoft, Paris, France). Pairwise associations among quantitative variables were assessed by bivariate analysis using two‐sided Pearson correlation coefficients.

Multivariate analyses included principal component analysis (PCA), agglomerated hierarchical clustering, and heatmap visualization. Principal component analysis was performed with XLSTAT using standardized values (mean = 0, standard deviation = 1) based on the correlation matrix. Agglomerated hierarchical clustering was conducted in XLSTAT after *z*‐standardization, using Ward's method and Euclidean distance. Heatmaps were generated in Microsoft Excel (version 2512, Microsoft Corporation, Redmond, WA, USA) from *z*‐standardized values to visualize co‐variation among parameters and sample groupings. Unless otherwise stated, the significance level was set to *α* = 0.05.

## RESULTS

### Baseline levels of total protein across harvest years, varieties, and regions

The baseline distribution of PPs across all malting barley malt samples is shown in Fig. 1, which presents boxplots of total protein, SN‐C, SN‐65, FAN, and KI by harvest year, barley variety, and regions of origin. Proteolytic parameters are influenced by numerous factors, including the mashing temperature profile and the duration of the mash rests.^45^ The congress mash, which promotes intensive proteolysis and cytolysis, primarily reflects the intrinsic proteolytic modification potential of the malt. In contrast, the 65 °C mash reflects nitrogen release under process‐relevant saccharification conditions.^11^, ^36^ Soluble nitrogen values from both mashing procedures were therefore included in the analysis.

**Figure 1:**
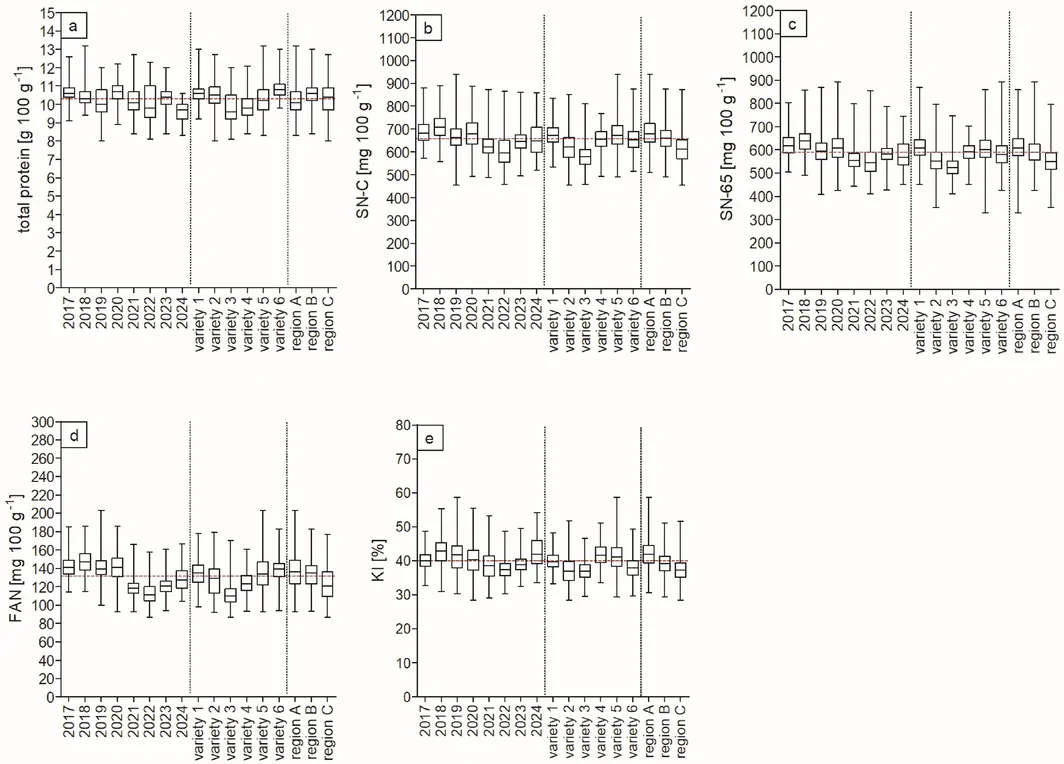
Box‐and‐whisker plots displaying (a) protein content, (b) soluble nitrogen in congress mash (SN‐C), (c) soluble nitrogen in 65 °C mash (SN‐65), (d) free amino nitrogen (FAN), and (e) Kolbach index (KI), separated by harvest year, barley variety, and region of origin (*n* = 7458 per factor). The horizontal line shows the overall mean across all groups.

As shown in Fig. 1, total protein content exhibited relatively moderate variability across most factors, although temporal and varietal effects contributed to discernible patterns within the dataset. The distribution for 2024 was noticeably narrower than those for the other harvest years, reflecting the smaller sample size for that year. Distributions for the remaining years were broader and generally similar in shape, indicating that total protein content was relatively stable under most conditions represented in the dataset. Temporal differences were nevertheless evident. As Table 1 reports, mean total protein levels ranged from 10.70 g 100 g^−1^ in 2017 to 9.60 g 100 g^−1^ in 2024, with standard deviations spanning 0.48 g 100 g^−1^ (2017) to 0.92 g 100 g^−1^ (2022). Although some years had similar mean values (e.g., 2019 and 2021), others, such as 2017 and 2024, differed by as much as 1.10 percentage points. These temporal differences are consistent with known year‐to‐year variation arising from environmental factors that influence sowing, nutrient availability, plant development, and fertilization practices.^18^, ^46^, ^47^

**Table 1:** Means and standard deviations of total protein content, soluble nitrogen in congress mash (SN‐C), soluble nitrogen in 65 °C mash (SN‐65), free amino nitrogen (FAN), and Kolbach index (KI) by harvest year (*n* = 7458)

Year	Sample size	Means/standard deviation									
		Total protein (g 100 g^−1^)		SN‐C (mg 100 g^−1^)		SN‐65 (mg 100 g^−1^)		FAN (mg 100 g^−1^)		KI (%)	
2017	531	10.70	0.48	685.89	49.29	620.15	51.08	142.05	11.03	40.10	2.52
2018	900	10.44	0.56	709.08	50.01	634.97	52.66	147.20	11.77	42.62	3.84
2019	1310	10.14	0.67	666.95	54.51	595.81	54.81	140.60	11.72	41.31	4.36
2020	1466	10.57	0.53	679.36	67.23	607.33	64.71	140.64	15.11	40.29	4.10
2021	1289	10.18	0.70	624.55	50.71	557.30	47.33	118.78	9.73	38.54	4.02
2022	959	10.08	0.92	603.73	64.63	549.35	58.54	112.45	10.86	37.50	2.63
2023	762	10.31	0.64	642.83	49.90	580.85	42.35	120.32	9.01	39.04	2.56
2024	241	9.60	0.53	652.51	72.85	577.68	58.38	128.01	12.89	42.51	4.19

Varietal differences in total protein content were more pronounced than regional differences, as shown in Fig. 1 and quantified in Table 2. Most barley varieties had mean total protein content above 10.00 g 100 g^−1^, with only varieties 3 (9.83 ± 0.75 g 100 g^−1^) and 4 (9.84 ± 0.60 g 100 g^−1^) falling below this threshold. The highest mean total protein content was observed for variety 6 (10.82 ± 0.44 g 100 g^−1^), followed by variety 1 (10.60 ± 0.70 g 100 g^−1^) and variety 2 (10.49 ± 0.77 g 100 g^−1^). Differences in median values between varieties are also evident in Fig. 1, with variety 3 exhibiting a median total protein content approximately 9% lower than that of variety 6. These results demonstrate substantial genotypic variation in protein content, and are further supported by the varietal clustering in Fig. 2, in which variety 3 forms a distinct cluster because of its consistently lower PP values.

**Table 2:** Means and standard deviations of total protein content, soluble nitrogen in congress mash (SN‐C), soluble nitrogen in 65 °C mash (SN‐65), free amino nitrogen (FAN), and Kolbach index (KI), by barley variety (*n* = 7458)

Variety	Sample size	Means/standard deviation									
		Total protein (g 100 g^−1^)		SN‐C (mg 100 g^−1^)		SN‐65 (mg 100 g^−1^)		FAN (mg 100 g^−1^)		KI (%)	
1	382	10.60	0.50	676.73	51.15	612.40	58.74	135.17	14.92	39.94	2.63
2	877	10.49	0.66	625.67	68.39	559.34	61.44	127.98	17.70	37.37	4.16
3	771	9.83	0.75	581.15	50.40	526.42	45.79	111.66	12.04	37.08	2.77
4	216	9.84	0.60	654.06	51.97	590.19	45.26	125.59	14.17	41.64	3.18
5	4609	10.27	0.67	675.27	59.23	605.33	55.85	134.96	16.04	41.23	3.83
6	603	10.82	0.44	653.84	53.54	581.79	55.27	137.00	13.27	37.86	3.08

**Figure 2:**
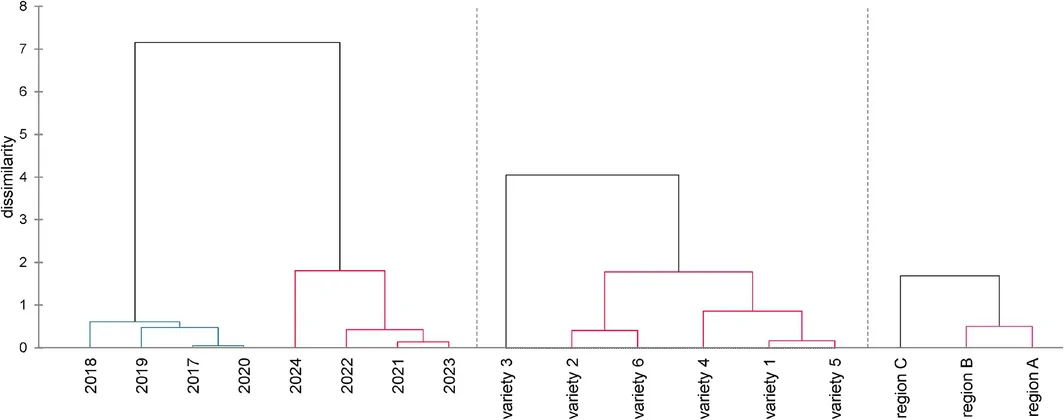
Dendrograms obtained by agglomerative hierarchical clustering (AHC) are shown separately by harvest year, barley variety, and region of origin, and are standardized to zero mean and unit variance. The clustering was performed using Ward's method and Euclidean distance, with cluster truncation determined using the Hartigan index.

Regional differences in total protein were comparatively limited but still detectable. As summarized in Table 3, mean total protein content ranged from 10.21 g 100 g^−1^ in region B to 10.54 g 100 g^−1^ in region A, with region C intermediate at 10.32 g 100 g^−1^. Standard deviations overlapped considerably among regions, indicating that geographical region contributed only modestly to variation in total protein content compared with the stronger effects of harvest year and variety. This pattern is reflected in the dendrogram in Fig. 2, in which samples did not cluster distinctively by region, and in the PCA results in Fig. 3, where regional categories appear scattered rather than forming discrete clusters.

**Table 3:** Means and standard deviations of total protein content, soluble nitrogen in congress mash (SN‐C), soluble nitrogen in 65 °C mash (SN‐65), free amino nitrogen (FAN), and Kolbach index (KI) by region of origin (*n* = 7458)

Region	Sample size	Means/standard deviation									
		Total protein (g 100 g^−1^)		SN‐C (mg 100 g^−1^)		SN‐65 (mg 100 g^−1^)		FAN (mg 100 g^−1^)		KI (%)	
A	3784	10.54	0.63	659.48	53.83	591.53	56.23	133.62	14.77	39.22	3.30
B	1247	10.21	0.66	683.99	57.54	612.62	55.49	136.47	16.05	42.00	3.67
C	2427	10.32	0.76	614.97	62.00	553.29	55.03	123.08	16.84	37.34	3.19

**Figure 3:**
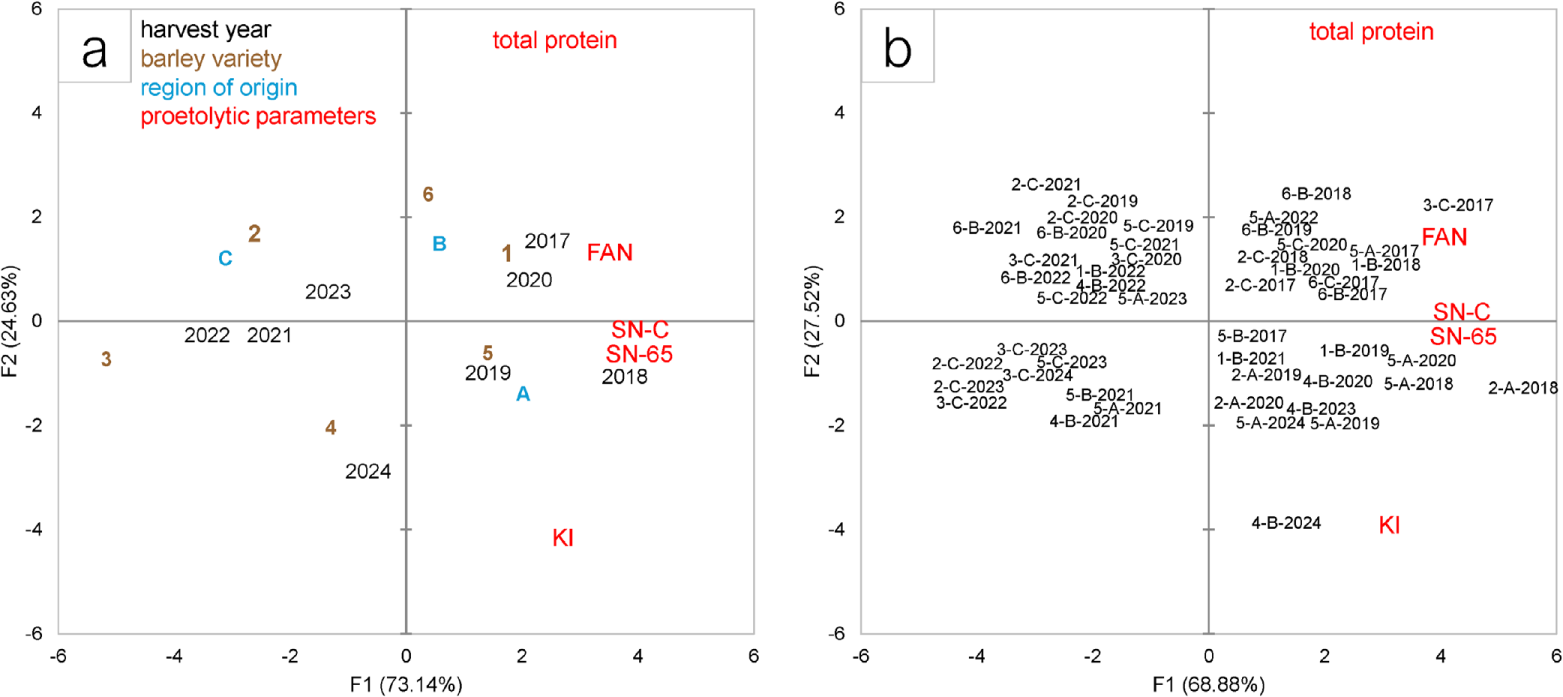
Principal component analysis (PCA) of protein characteristics. Panel (a) displays the distribution of samples by mean values for harvest year, barley variety, and region of origin, accounting for 97.77% of the total variance. Panel (b) displays the distribution of samples for each ID, which combines variety, region, and year, accounting for 94.6% of the total variance.

The distribution patterns shown in Fig. 1 are further illustrated in Fig. 4, which presents standardized PP values by harvest year for each variety and region. For total protein, the heatmap indicates that values fluctuate both positively and negatively across years without a consistent temporal trend. In contrast to SN and FAN, which show clear declines over time, total protein exhibits variety‐dependent and region‐dependent fluctuations. For example, in 2022, varieties 4 and 6, both originating from the same region, showed markedly different total protein contents, highlighting strong variety × year interaction. This interaction is quantified in Table 4, where the partial eta‐squared values show that the variety × year interaction (*η*p^2^ = 0.367) exceeds those for the main effect of harvest year (*η*p² = 0.218) and the region × year interaction (*η*p² = 0.217). These higher order interactions are consistent with the heatmap in Fig. 4, in which annual deviations in total protein appear idiosyncratic across varieties and regions.

**Figure 4:**
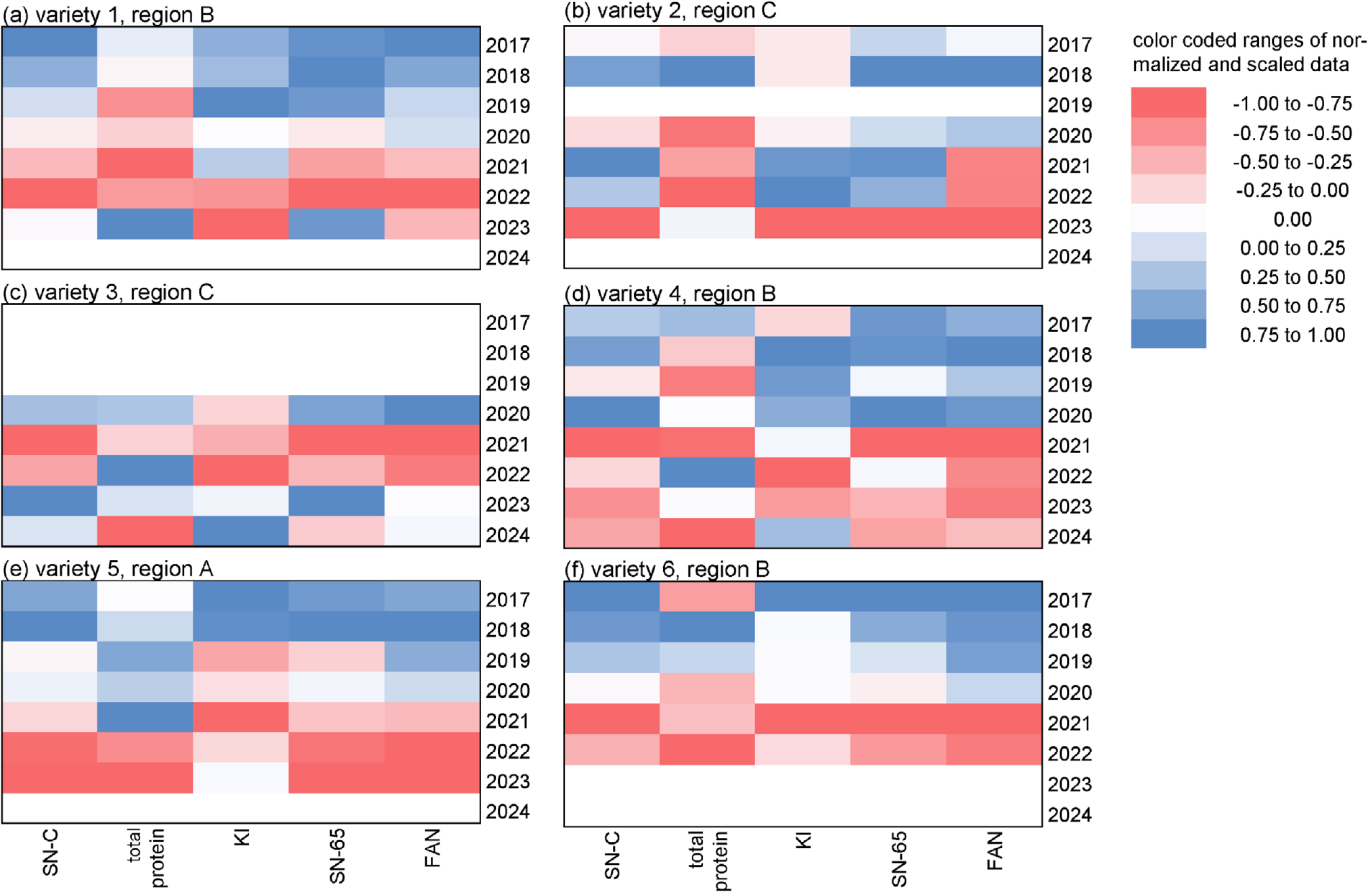
Heatmaps of protein content, soluble nitrogen in congress mash (SN‐C), free amino nitrogen (FAN), Kolbach index (KI), and soluble nitrogen in 65 °C mash (SN‐65) for all varieties across their respective harvest years are displayed for each of their main region of origin. Panel (a) shows variety 1 from region B, panel (b) shows variety 2 from region C, panel (c) shows variety 3 from region C, panel (d) shows variety 4 from region B, panel (e) shows variety 5 from region A, and panel (f) shows variety 6 from region B. Data were standardized to a mean of 0 and a variance of 1, and scaled from −1 to 1, with colored intervals of 0.25.

**Table 4:** Effect sizes (partial eta‐squared) for main effects and two‐ and three‐way interactions of harvest year, barley variety, and region of origin impacting total protein content, soluble nitrogen in congress mash (SN‐C), soluble nitrogen in 65 °C mash (SN‐65), free amino nitrogen (FAN) and Kolbach index (KI); strongest effects per variable are highlighted in bold

PPs	Partial eta‐squared (*η*p^2^)						
	Year	Variety	Region	Year × variety	Year × region	Variety × region	Year × variety × region
Protein	0.218	0.178	0.041	**0.367**	0.217	0.006	0.003
SN‐C	**0.321**	0.202	0.071	0.071	0.040	0.001	0.002
SN‐65	**0.265**	0.181	0.054	0.052	0.037	0.001	0.002
FAN	**0.587**	0.121	0.023	0.063	0.036	0.001	0.002
KI	**0.244**	0.262	0.123	0.129	0.051	0.001	0.002

The combined‐ID PCA plot in Fig. 3(b) provides a more detailed representation of the simultaneous effects of variety, region of origin, and harvest year. Combined IDs from earlier harvest years (e.g., 3‐C‐2017 and 2‐A‐2018) are located on the positive side of F1, whereas those from later years (e.g., 5‐A‐2024 and 2‐C‐2023) cluster on the negative side. Total protein contributes to this separation but is not the primary differentiating variable. Instead, it contributes jointly with SN and FAN to the overall multivariate structure.

### Variety effects and classification of modification levels

Varietal differences were a major source of variation within the dataset, particularly for SN (SN‐C and SN‐65), FAN, and KI, while exerting comparatively smaller, yet still detectable, effects on total protein. These distinctions are clearly illustrated in Fig. 1, which shows the distribution of PPs by barley variety, and are further supported by the quantitative summaries in Table 2, which present the mean and standard deviation of each parameter for each variety. The clustering results in Fig. 2 also demonstrate that variety is a major determinant of PP patterns, particularly in comparison with region of origin.

Across all varieties, SN showed the strongest varietal influence. The highest absolute values were observed for varieties 1 and 5, with SN‐C values of 676.73 and 675.27 mg 100 g⁻¹, respectively, and SN‐65 values of 612.40 and 605.33 mg 100 g⁻¹. Both varieties consistently exceeded the overall dataset mean and displayed broad distributions in Fig. 1 that were shifted upward relative to those of the other varieties. Varieties 2, 4, and 6 occupied an intermediate range, with SN‐C values of 625.67–654.06 mg 100 g⁻¹ and SN‐65 values of 559.34–590.19 mg 100 g⁻¹. Variety 3 exhibited the lowest SN values, with an SN‐C of 581.15 mg 100 g⁻¹ and an SN‐65 of 526.42 mg 100 g⁻¹, and showed the narrowest distributions at the lowest positions in Fig. 1

These SN levels correspond closely to the FAN levels. As Fig. 1 and Table 2 show, varieties 1, 5, and 6 exhibited the highest FAN values, ranging from 134.96 to 137.00 mg 100 g^−1^, consistent with their SN levels. These varieties also showed clearly separated median FAN values and narrower distributions than the remaining varieties. Varieties 2 (127.98 mg 100 g^−1^) and 4 (125.59 mg 100 g^−1^) formed an intermediate FAN group with broader, overlapping distributions that nevertheless remained consistently below those of the high‐FAN varieties. Variety 3 (111.66 mg 100 g^−1^) consistently exhibited the lowest FAN values across all harvest years and regions, forming a distinct low‐FAN group. The distributions shown in Fig. 1 closely reflect these groupings, with clear separation between varieties 1, 5, and 6, varieties 2 and 4, and particularly 3.

Variety 6 showed an important deviation from the overall pattern. Despite intermediate SN values, it exhibited the highest FAN values of all varieties. This pattern is evident in Fig. 1, where the median FAN value for variety 6 exceeds those of varieties 1 and 5 despite its lower SN‐C and SN‐65 values. This discrepancy indicates that FAN formation, although closely associated with SN content, is not determined solely by it. The relationship between SN and FAN therefore appears to vary among varieties, with variety 6 exhibiting disproportionately high FAN formation relative to its SN content. This pattern is reflected in the PCA (Fig. 3(a)), in which FAN has a strong loading and samples of variety 6 have high F1 scores, and in the combined‐ID PCA (Fig. 3(b)), where many identifiers containing variety 6 cluster toward the high‐FAN quadrant.

The modification classification was defined *a priori* based on expert evaluation and long‐term malting experience and reflects the established malting behavior of the varieties. It comprised the two groups: a high‐modification group consisting of varieties 1, 4, and 5, and a moderate‐modification group consisting of varieties 2, 3, and 6. The KI results (Table 2) support this classification. Varieties 1 (39.94%), 4 (41.64%), and 5 (41.23%) consistently exhibited higher KI values, consistent with the high‐modification group, whereas varieties 2 (37.37%), 3 (37.08%), and 6 (37.86%) exhibited lower KI values consistent with the moderate‐modification group.

The heatmap representation in Fig. 4 provides additional insight into variety‐specific temporal patterns. Across varieties, FAN, SN‐C, and SN‐65 values declined consistently during the later harvest years (2021–2024). Although differences among varieties persisted throughout the study period, their magnitude was reduced in the later years. A few exceptions were observed, particularly for varieties 1 and 3 in specific region × year combinations, where declines in SN were less pronounced. These exceptions indicate that varietal characteristics modulate harvest year effects but do not override them. The total protein content in Fig. 4 also reinforces earlier observations: unlike SN and FAN, total protein does not show a consistent temporal trend but instead reflects a strong harvest year × variety interaction.

Variety‐driven differences were also evident in the hierarchical clustering (Fig. 2, center panel). Variety 3 formed a distinct cluster, separated from all other varieties, consistent with its low protein, low SN, and low FAN values. The remaining varieties were divided into two clusters: one comprising varieties 2 and 6, which generally exhibited moderate modification characteristics, and a second comprising varieties 1, 4, and 5, which shared a high modification profile.

### Harvest year effects on SN and FAN

Harvest year was the strongest individual factor influencing SN (SN‐C, SN‐65) and FAN levels across the dataset. This dominant effect is consistently reflected in the visual and numerical summaries, including the boxplots in Fig. 1, the dendrogram in Fig. 2, the heatmap in Fig. 4, the PCA in Fig. 3, and the ANOVA effect sizes in Table 4. Whereas the total protein content exhibited only moderate temporal fluctuations, SN and FAN showed pronounced systematic changes across years, particularly between 2017–2020 and 2021–2024.

The contrast between the earlier and later harvest years was most evident for the SN parameters. As summarized in Table 1, mean SN‐C values during 2017–2020 ranged from moderate to high and frequently exceeded 650 mg 100 g⁻¹, whereas mean values during 2021–2024 were consistently lower and more variable. A similar pattern was observed for SN‐65, which also declined during 2021–2024. The greater variability observed in 2020 and 2024 for both SN parameters, reflected by larger standard deviations, indicates more heterogeneous raw material conditions in those years. This heterogeneity is also visible in Fig. 1, where the spreads for SN‐C and SN‐65 in these years are visibly broader in 2020 and 2024 than in the remaining harvest years.

The downward shift in SN over time is most strongly reflected in FAN, which displayed the clearest and most consistent temporal decline among all PPs. As Table 1 reports, mean FAN values decreased from 147.20 ± 11.77 mg 100 g^−1^ in 2018 to 112.45 ± 10.86 mg 100 g^−1^ in 2022, representing an absolute reduction of 34.75 mg 100 g^−1^ or 23.6%. Intermediate years displayed FAN levels between 140.72 ± 11.72 mg 100 g^−1^ and 128.01 ± 12.89 mg 100 g^−1^, indicating a gradual decline rather than abrupt changes. The temporal decrease in FAN is clearly shown in Fig. 1, where FAN distributions for early years are shifted upwards relative to later years. Figure 4 also shows this decline across nearly all varieties and regions; the heatmap displays increasingly negative‐scaled FAN values in the lower rows corresponding to the later years. Only minor deviations from this pattern occur, such as for variety 1 in region B in 2023 and variety 3 in isolated years, but these instances do not alter the overall temporal trend.

Harvest year‐based clustering in Fig. 2 further supports these observations. The dendrogram (left panel) separates the dataset into two main clusters, comprising harvest years 2017–2020 and 2021–2024, respectively. Within the earlier cluster, 2018 forms a distinct branch, whereas within the later cluster, 2024 diverges from 2021–2023, likely due to its narrower distributions and higher variability in SN values. Despite these within‐cluster differences, the overarching two‐cluster structure remained consistent, demonstrating that harvest year was the dominant source of variation among PPs.

The temporal separation observed in the boxplots and cluster analysis is also evident in the PCA results (Fig. 3). In the PCA biplot (panel a), samples from 2017–2020 cluster on the positive F1 side, aligned with high loadings for total protein, FAN, SN‐C, SN‐65, and KI. Conversely, samples from 2021–2024 are located predominantly occupy the negative F1 region. The strong association of SN and FAN with the positive direction of F1, combined with the temporal grouping indicates that declining SN is the primary driver of PCA structure. Harvest year, therefore, explains a substantial portion of the PP variation, as indicated by ANOVA results in Table 4, where the main effect of year for FAN is *η*p^2^ = 0.587, the largest effect size across all traits.

The combined‐ID PCA (Fig. 3(b)) offers additional detail by illustrating how harvest year interacts with variety and region. Identifiers corresponding to the earlier harvest years (e.g., 3‐C‐2017, 2‐A‐2018) cluster consistently on the right side of F1, whereas those corresponding to later years (e.g., 2‐C‐2023, 5‐A‐2024) cluster on the left side. This spatial separation confirms that harvest year is the dominant determinant of PP patterns regardless of varietal or regional grouping.

Temporal effects are also evident within individual varieties and regions. The heatmap in Fig. 4 shows that the effects of harvest year on SN‐C, SN‐65, and FAN are consistent across nearly all varieties and regions. Across the vertical axis (years), the progression from predominantly blue/white shading (higher or average values) in early years to predominantly red shading (lower values) in later years illustrates the systematic decline in nitrogen‐related parameters. Boxplots (Fig. 1), dendrograms (Fig. 2), heatmaps (Fig. 4), PCA (Fig. 3), and ANOVA results (Table 4) consistently identify harvest year as the dominant factor influencing SN and FAN. Earlier harvest years are associated with higher SN and FAN values, whereas later harvest years consistently exhibit lower values, indicating that harvest year is the primary determinant of PP distributions across the dataset.

### Regional effects and factor‐adjusted interactions among PPs

Regional influences on PPs were consistently smaller than those of harvest year and barley variety. As Fig. 1 shows, the regional distributions of total protein, SN, FAN, and KI overlapped, with only modest differences in central tendency. The quantitative summaries in Table 3 likewise show that all regions fall within relatively narrow ranges for each parameter, indicating that geographic region exerted a measurable but secondary influence on PPs.

Across regions A, B, and C, mean total protein content ranged from 10.21 g 100 g^−1^ in region B to 10.54 g 100 g^−1^ in region A, with region C at 10.32 g 100 g^−1^. The overlapping standard deviations (0.63–0.74 g 100 g^−1^) further indicate that the regional effects on protein content were modest. The ranking of regional means differs between total protein and SN/FAN. Although region A had the highest total protein content, region B produced the highest average SN‐C, SN‐65, and FAN values. The SN ranged from 614.97 ± 62.00 mg 100 g^−1^ (region C) to 683.99 ± 57.54 mg 100 g^−1^ (region B), with region A occupying an intermediate position. The FAN values ranged from 123.08 ± 16.84 mg 100 g^−1^ (region C) to 136.47 ± 16.05 mg 100 g^−1^ (region B). In contrast, KI showed limited regional differentiation, with means across regions ranging from 37.34 ± 3.19% to 42.00 ± 3.67%.

The hierarchical clustering shown in Fig. 2 further supports these findings. The right‐hand dendrogram, representing regional groupings, shows only minor separation, with no distinct clusters among the three regions. The small distances between regional branches in the dendrogram indicate that the region contributes relatively little to PP differentiation. This contrasts with the clear separations observed in the dendrograms for harvest year and variety, which show larger clusters, illustrating the relative importance of each factor and emphasizing the comparatively weak influence of geographic region on proteolytic quality parameters.

The magnitude of regional effects is further clarified through the ANOVA effect sizes reported in Table 4. Across all PPs, the main effect of region is consistently smaller than the effects of harvest year and variety. Interactions involving the region of origin, particularly variety × region and harvest year × region, display very small effect sizes (the smallest and second smallest among all two‐way interactions). The three‐way year × variety × region interaction is negligible across all PPs. This pattern indicates that regional differences, while present, do not substantially modulate the underlying variety‐ or harvest‐year‐driven behavior of PPs.

Correlation structures also reveal limited regional influence. As Table 5 reports, correlation coefficients between total protein and SN remained stable across regions, ranging from *r* = 0.300 (region C) to *r* = 0.318 (region A). This narrow bandwidth suggests that the relationship between total protein and SN is consistent across geographic regions. By comparison, year‐based correlations ranged widely (from −0.08 in 2018 to 0.762 in 2022), demonstrating much stronger temporal modulation. Correlations between SN‐C and SN‐65 remained consistently high across regions (r = 0.756–0.938), and SN–FAN correlations also remained strong and positive, mirroring the global dataset structure. Strong relationships among these nitrogen‐related traits across regions indicate that the biochemical interdependence between SN and FAN formation was stable regardless of the region of origin.

**Table 5:** Pearson correlation coefficients for correlations between total protein content, soluble nitrogen in congress mash (SN‐C), soluble nitrogen in 65 °C mash (SN‐65), free amino nitrogen (FAN), and Kolbach index (KI) (rows) and harvest years, barley varieties, and regions of origin (columns); significant correlations (*P* < 0.05) are highlighted in bold

Factors	Pearson correlation coefficient (*r*)								
	Protein × SN‐C	Protein × SN‐65	Protein × FAN	Protein × KI	SN‐C × SN‐65	SN‐C × FAN	SN‐C × KI	SN‐65 × FAN	FAN × KI
All data	**0.322**	**0.287**	**0.314**	**−0.339**	**0.901**	**0.895**	**0.776**	**0.812**	**0.683**
2017	**0.496**	**0.461**	**0.394**	**−0.130**	**0.853**	**0.959**	**0.796**	**0.796**	**0.819**
2018	**−0.080**	**−0.087**	−0.048	**−0.614**	**0.840**	**0.968**	**0.825**	**0.818**	**0.786**
2019	**−0.056**	**−0.145**	**0.105**	**−0.644**	**0.888**	**0.926**	**0.797**	**0.796**	**0.648**
2020	**0.211**	**0.173**	**0.211**	**−0.310**	**0.870**	**0.877**	**0.858**	**0.789**	**0.747**
2021	0.004	0.043	**0.160**	**−0.635**	**0.828**	**0.867**	**0.758**	**0.747**	**0.561**
2022	**0.762**	**0.729**	**0.721**	**−0.137**	**0.938**	**0.982**	**0.532**	**0.929**	**0.560**
2023	**0.578**	**0.508**	**0.502**	**−0.258**	**0.892**	**0.975**	**0.635**	**0.877**	**0.676**
2024	**0.482**	**0.460**	**0.486**	−0.029	**0.936**	**0.996**	**0.861**	**0.936**	**0.853**
Variety 1	**0.517**	**0.350**	**0.527**	−0.094	**0.756**	**0.931**	**0.793**	**0.730**	**0.723**
Variety 2	**0.239**	**0.180**	**0.301**	**−0.310**	**0.891**	**0.925**	**0.847**	**0.824**	**0.738**
Variety 3	**0.564**	**0.469**	**0.637**	**−0.352**	**0.882**	**0.895**	**0.567**	**0.810**	**0.380**
Variety 4	**0.455**	**0.408**	**0.456**	**−0.329**	**0.873**	**0.880**	**0.687**	**0.789**	**0.550**
Variety 5	**0.281**	**0.279**	**0.169**	**−0.428**	**0.882**	**0.873**	**0.740**	**0.785**	**0.701**
Variety 6	**0.277**	**0.187**	**0.289**	**−0.211**	**0.920**	**0.900**	**0.869**	**0.820**	**0.779**
Region A	**0.318**	**0.322**	**0.139**	**−0,414**	**0.877**	**0.894**	**0.725**	**0.806**	**0.752**
Region B	**0.337**	**0.220**	**0.505**	**−0.386**	**0.861**	**0.858**	**0.727**	**0.721**	**0.480**
Region C	**0.300**	**0.260**	**0.403**	**−0.283**	**0.855**	**0.786**	**0.826**	**0.700**	**0.553**

The KI correlations likewise showed little regional differentiation. The KI was consistently positively correlated with SN and FAN across all regions and negatively correlated with total protein. No substantial deviations emerged in any region or harvest year combination, except for a slightly stronger negative correlation in 2021 (*r* = −0.635), which reflects a general dataset pattern rather than a region‐specific anomaly.

Together, these integrated results demonstrate that regional effects, although measurable, played a secondary role in shaping PPs compared with harvest year and variety. Regional differences appeared primarily as subtle shifts in magnitude rather than as major drivers of pattern formation. Across multivariate, cluster‐based, and correlation‐based analyses, geographic region made only a minor contribution to PP variability, underscoring its position as the least influential of the three factors that were investigated.

## DISCUSSION AND CONCLUSION

The findings of this study are most coherently interpreted within the sequential nitrogen cascade linking total protein, varietal modification potential, SN, and FAN formation (Fig. 5). Total protein is the biochemical starting point for this pathway and shows moderate variation across varieties and years. Its relative temporal stability is consistent with earlier reports showing that grain protein accumulation depends primarily on genotype, nitrogen availability, and grain‐filling conditions, rather than year‐to‐year climatic variability.^7^, ^48^ The pronounced varietal differences observed in the present study, ranging from the consistently low protein levels of variety 3 to the substantially higher levels of variety 6, identified genotype as a major determinant of protein accumulation and upstream nitrogen potential.^18^, ^35^

**Figure 5:**
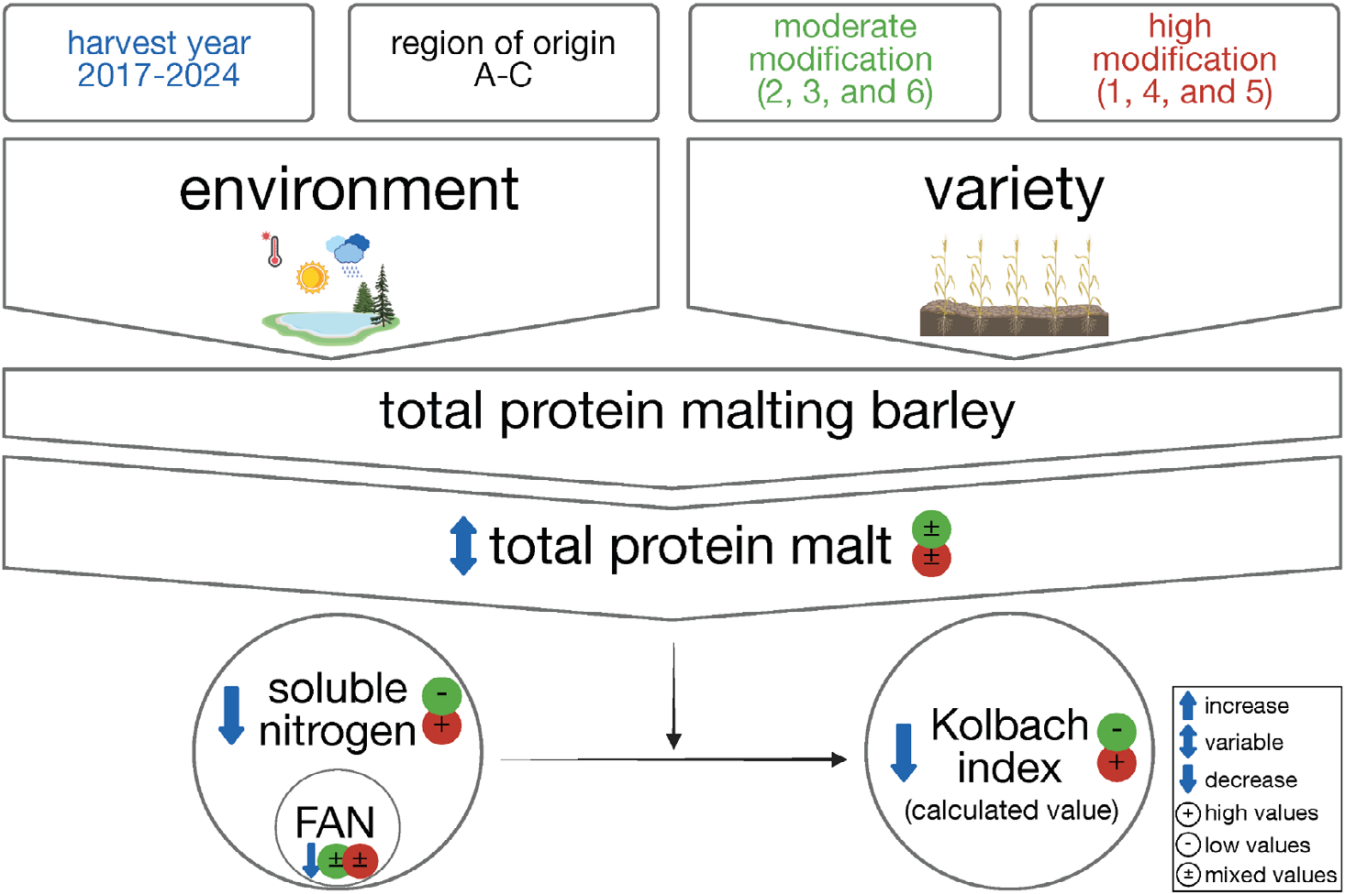
Schematic representation showing how environmental factors (harvest year, region of origin) and varietal characteristics (degree of modification) influence total protein content in barley and malting barley, which contributes to soluble nitrogen (SN) with free amino nitrogen (FAN) as a constituent fraction and the calculated Kolbach index (KI). Created with BioRender.com.

High SN levels were consistent with the high FAN values observed in varieties 1 and 5, whereas variety 3 consistently exhibited low values for both parameters. Variety 6 displayed a distinctive profile: it produced the highest FAN values despite only moderate SN levels. Similar genotype‐specific differences between protein modification and FAN generation have been reported in multi‐environment proteomic studies, in which enhanced FAN production was associated with genotypes with favorable protease isoforms or endosperm degradation dynamics.^20^, ^22^, ^49^ These results indicate that FAN generation is not determined solely by SN content but also reflects genetically anchored proteolytic efficiency.

Within this cascade, SN serves as the central biochemical intermediate, and its strong correlation with FAN in the present dataset is consistent with its established role in amino nitrogen release during germination.^45^, ^50^ Unlike total protein, SN showed a pronounced temporal decline, especially during the 2021–2024 harvest years. This pattern reflects shifts in protein modification efficiency, which is sensitive to climatic and stress factors such as heat, drought, or altered nitrogen remobilization during grain maturation.^11^, ^23^, ^26^, ^33^ The parallel decline in FAN indicates that the environmental conditions in the recent harvest years affected the modification stage of the cascade more strongly than protein accumulation itself.

Free amino nitrogen was also highly responsive to environmental conditions, with higher values during the earlier harvest years (2017–2020) followed by a steady decline. This pattern is consistent with previous studies showing that FAN responds sensitively to environmental conditions.^7^, ^36^ The large proportion of FAN variance explained by harvest year in this study is consistent with multi‐environment analyses, demonstrating that year‐to‐year environmental variation often exceeds genetic or locational effects for nitrogen‐related malt characteristics.^21^, ^22^ The decline in FAN despite variable total protein content further indicates that environmental conditions primarily influence protein quality rather than protein quantity.

Regional effects were present but comparatively small. Although region B tended to exhibit higher SN and FAN values, these differences did not substantially alter the overall structure of the nitrogen pathway. Similar observations have been reported for barley grown under different regional conditions, where geographic effects generally remain modest unless climatic regimes differ markedly.^6^, ^49^, ^51^

The KI, which represents the ratio of soluble to total nitrogen, showed comparatively little variation across varieties, harvest years, and regions of origin. This stability reflects its nature as a proportional measure. It is consistent with reports that KI is reliable primarily when total protein levels fall within conventional malting ranges and when malting is conducted to a defined SN specification but is less sensitive to shifts in absolute SN.^7^, ^50^ In the present dataset, KI remained within a narrow range and did not show the pronounced temporal decline observed for SN and FAN. Instead, KI reflected the expert‐defined modification classification: varieties 1, 4, and 5 consistently showed higher KI values, corresponding to the high‐modification group, whereas varieties 2, 3, and 6 showed lower KI values, consistent with the moderate‐modification group.

The results demonstrate that nitrogen‐related malt quality traits follow a sequential cascade in which total protein defines the initial substrate, barley variety determines the inherent modification potential, harvest year modulates the efficiency of SN formation, and FAN reflects the resulting proteolytic conversion capacity. Regional influences are secondary. The temporal decline in SN and FAN in the more recent years, despite differing protein levels, highlights the importance of evaluating both protein quantity and protein quality. Grouping varieties into modification classes provides a useful framework for integrating genetic predisposition with environmental responsiveness. This cascade‐oriented perspective offers a robust basis for interpreting quality variation in malting barley across production environments and supports informed decisions in variety selection, raw‐material sourcing, and malt quality assessment.

## FUNDING INFORMATION

This project was supported by the Joint Science Conference, the Federal Ministry of Education and Research (BMBF), the State of Rhineland‐Palatinate, and the Nikolaus Koch Foundation.

## CONFLICT OF INTEREST

The authors declare no conflict of interest.

## AUTHOR CONTRIBUTIONS

Shari Piro: conceptualization; data curation; formal analysis; investigation; methodology; writing – original draft. Martina Gastl: conceptualization; investigation; methodology; project administration; writing – review and editing. Michael Fechir: conceptualization; investigation; methodology; project administration; validation; writing – review and editing.

## Supporting information


**Table S1.** Means and standard deviations of total protein content, soluble nitrogen in congress mash (SN‐C), soluble nitrogen in 65 °C mash (SN‐65), free amino nitrogen (FAN), and Kolbach index (KI) by ID comprising variety, region of origin, and harvest year (n = 7458).

## Data Availability

The data that support the findings of this study are available from the corresponding author upon reasonable request.
