# Supplementary material for: Impact of variety and environmental factors on proteolytic quality parameters of selected malting barley varieties
Source: J Sci Food Agric. 2026 Jul 7;106(14):8628–38. doi: 10.1002/jsfa.70866 (PMC13651928; doi:10.1002/jsfa.70866)
Supplement: Supplementary file 1 [file JSFA-106-8628-s001.docx]

Supporting Information

IDs for variety, region of origin, and harvest year, along with their respective sample sizes (n). For all measured parameters, including total protein, SN-C, SN-65, FAN, and KI, means and standard deviations are displayed. IDs with a sample size of n ≥ 10 are bolded, as only these were included for ID-based analyses. It is evident that each variety is predominantly associated with a single region of origin. Furthermore, not all varieties were included across all harvest years in the dataset, as raw material availability fluctuated over the years. This presentation allows for a clear distinction between adequately represented groups and smaller groups (n < 10), which were excluded from further ID-based analysis. Thus, IDs with a sample size of n ≥ 10 are highlighted in bold.

**Table S-1**

| ID | sample  size | mean and standard deviation | | | | | | | | | | | | | |
| --- | --- | --- | --- | --- | --- | --- | --- | --- | --- | --- | --- | --- | --- | --- | --- |
|  |  | total protein  [g 100 g^-1^] | |  | SN-C  [mg 100 g^-1^] | |  | SN-65  [mg 100 g^-1^] | |  | FAN  [mg 100 g^-1^] | |  | KI  [%] | |
| 1-B-2017 | 6 | 10.98 | 0.39 |  | 718.67 | 57.37 |  | 640.50 | 40.62 |  | 147.50 | 14.65 |  | 40.88 | 2.72 |
| **1-B-2018** | 101 | 10.86 | 0.61 |  | 705.63 | 43.33 |  | 642.53 | 37.75 |  | 143.84 | 11.41 |  | 40.65 | 2.14 |
| **1-B-2019** | 37 | 10.35 | 0.31 |  | 688.08 | 48.11 |  | 638.46 | 73.89 |  | 137.57 | 9.59 |  | 41.55 | 2.82 |
| **1-B-2020** | 149 | 10.68 | 0.34 |  | 672.34 | 51.13 |  | 602.89 | 66.48 |  | 136.65 | 14.74 |  | 39.44 | 2.65 |
| **1-B-2021** | 53 | 10.15 | 0.37 |  | 654.43 | 36.87 |  | 586.28 | 33.92 |  | 124.85 | 920 |  | 40.32 | 2.48 |
| **1-B-2022** | 34 | 10.40 | 0.34 |  | 626.12 | 32.48 |  | 571.44 | 27.04 |  | 114.32 | 6.12 |  | 37.57 | 1.74 |
| 1-B-2023 | 1 | 11.50 | - |  | 676.00 | - |  | 639.00 | - |  | 124.00 | - |  | 36.80 | - |
| 1-C-2020 | 1 | 10.10 | - |  | 645.00 | - |  | 602.00 | - |  | 141.00 | - |  | 39.90 | - |
| 2-A-2017 | 1 | 10.40 | - |  | 651.00 | - |  | 603.00 | - |  | 130.00 | - |  | 39.10 | - |
| **2-A-2018** | 53 | 10.36 | 0.18 |  | 762.70 | 32.15 |  | 686.83 | 24.86 |  | 159.81 | 7.11 |  | 46.04 | 2.18 |
| **2-A-2019** | 44 | 9.95 | 0.41 |  | 651.09 | 50.51 |  | 584.93 | 43.85 |  | 133.37 | 9.64 |  | 40.90 | 2.49 |
| **2-A-2020** | 18 | 9.71 | 0.34 |  | 645.56 | 21.07 |  | 578.22 | 19.50 |  | 128.94 | 4.12 |  | 41.62 | 1.87 |
| 2-B-2020 | 1 | 9.40 | - |  | 647.00 | - |  | 584.00 | - |  | 129.00 | - |  | 43.00 | - |
| **2-C-2017** | 112 | 10.40 | 0.32 |  | 649.14 | 41.96 |  | 580.84 | 37.10 |  | 134.89 | 9.02 |  | 39.03 | 2.22 |
| 2-C-2018 | 95 | 10.61 | 0.40 |  | 659.41 | 45.12 |  | 585.19 | 46.04 |  | 138.10 | 9.60 |  | 38.95 | 3.21 |
| **2-C-2019** | 131 | 10.92 | 0.54 |  | 614.49 | 45.05 |  | 539.44 | 41.80 |  | 134.21 | 9.38 |  | 35.20 | 2.34 |
| **2-C-2020** | 153 | 10.68 | 0.47 |  | 621.30 | 75.77 |  | 555.10 | 73.95 |  | 128.66 | 20.23 |  | 36.35 | 4.01 |
| **2-C-2021** | 132 | 11.09 | 0.56 |  | 601.24 | 48.55 |  | 535.52 | 32.44 |  | 116.31 | 8.54 |  | 33.95 | 3.28 |
| **2-C-2022** | 120 | 9.64 | 0.44 |  | 558.35 | 36.42 |  | 510.52 | 35.20 |  | 105.68 | 6.39 |  | 36.22 | 2.14 |
| **2-C-2023** | 17 | 9.40 | 0.16 |  | 555.94 | 24.89 |  | 506.65 | 28.73 |  | 105.63 | 4.73 |  | 37.02 | 1.77 |
| 3-B-2019 | 1 | 9.40 | - |  | 694.00 | - |  | 615.00 | - |  | 152.00 | - |  | 46.10 | - |
| **3-C-2017** | 11 | 11.52 | 0.36 |  | 740.55 | 51.91 |  | 661.09 | 48.64 |  | 155.36 | 10.91 |  | 40.17 | 2.35 |
| **3-C-2020** | 33 | 10.46 | 0.47 |  | 620.21 | 43.47 |  | 562.58 | 67.04 |  | 136.42 | 9.06 |  | 37.19 | 2.62 |
| **3-C-2021** | 277 | 10.41 | 0.49 |  | 596.91 | 40.90 |  | 536.22 | 38.80 |  | 115.14 | 8.08 |  | 35.94 | 2.75 |
| **3-C-2022** | 357 | 9.29 | 0.33 |  | 558.67 | 39.46 |  | 509.14 | 37.15 |  | 104.97 | 7.21 |  | 37.66 | 2.67 |
| **3-C-2023** | 70 | 9.73 | 1.00 |  | 587.38 | 57.30 |  | 535.70 | 42.96 |  | 111.22 | 10.20 |  | 37.80 | 1.83 |
| **3-C-2024** | 22 | 9.72 | 0.23 |  | 584.27 | 38.78 |  | 528.36 | 33.06 |  | 116.85 | 7.76 |  | 37.56 | 1.95 |
| **4-B-2020** | 65 | 10.08 | 0.48 |  | 673.77 | 48.17 |  | 608.97 | 49.42 |  | 138.66 | 13.55 |  | 41.80 | 2.49 |
| **4-B-2021** | 66 | 9.51 | 0.45 |  | 614.56 | 48.77 |  | 559.92 | 39.63 |  | 113.58 | 8.72 |  | 40.47 | 3.05 |
| **4-B-2022** | 23 | 10.40 | 0.56 |  | 632.13 | 33.39 |  | 576.74 | 35.43 |  | 115.52 | 6.32 |  | 38.01 | 1.48 |
| **4-B-2023** | 51 | 9.91 | 0.53 |  | 687.79 | 31.12 |  | 613.45 | 25.64 |  | 128.31 | 6.22 |  | 43.45 | 2.11 |
| **4-B-2024** | 11 | 8.86 | 0.10 |  | 664.06 | 24.87 |  | 581.16 | 23.45 |  | 128.83 | 4.82 |  | 46.83 | 1.96 |
| **5-A-2017** | 259 | 10.90 | 0.47 |  | 703.52 | 46.09 |  | 640.83 | 50.10 |  | 144.88 | 11.03 |  | 40.38 | 2.71 |
| **5-A-2018** | 550 | 10.20 | 0.37 |  | 718.25 | 44.81 |  | 642.48 | 50.66 |  | 149.04 | 11.15 |  | 44.09 | 2.98 |
| **5-A-2019** | 806 | 9.74 | 0.40 |  | 680.80 | 51.91 |  | 611.61 | 48.55 |  | 142.30 | 12.46 |  | 43.72 | 3.13 |
| **5-A-2020** | 545 | 10.50 | 0.65 |  | 724.81 | 51.40 |  | 645.18 | 50.54 |  | 147.64 | 12.71 |  | 43.25 | 3.19 |
| **5-A-2021** | 488 | 9.67 | 0.37 |  | 640.14 | 44.96 |  | 565.69 | 47.06 |  | 120.05 | 9.35 |  | 41.41 | 2.89 |
| **5-A-2022** | 267 | 11.21 | 0.45 |  | 675.42 | 46.19 |  | 610.96 | 44.91 |  | 123.62 | 8.38 |  | 37.73 | 2.91 |
| **5-A-2023** | 547 | 10.50 | 0.38 |  | 653.16 | 37.41 |  | 588.25 | 34.24 |  | 122.00 | 7.04 |  | 38.92 | 2.33 |
| **5-A-2024** | 206 | 9.62 | 0.55 |  | 659.67 | 73.80 |  | 583.15 | 59.53 |  | 129.23 | 13.10 |  | 42.84 | 4.01 |
| **5-B-2017** | 19 | 10.29 | 0.47 |  | 649.95 | 40.63 |  | 581.79 | 32.41 |  | 130.05 | 8.17 |  | 39.47 | 1.60 |
| 5-B-2018 | 4 | 10.23 | 0.13 |  | 627.00 | 21.80 |  | 563.00 | 25.85 |  | 125.25 | 4.35 |  | 38.30 | 0.96 |
| 5-B-2019 | 1 | 9.80 | - |  | 632.00 | - |  | 586.00 | - |  | 126.00 | - |  | 40.30 | - |
| 5-B-2020 | 8 | 10.73 | 0.32 |  | 705.88 | 44.87 |  | 635.25 | 45.78 |  | 145.69 | 6.61 |  | 41.14 | 1.96 |
| **5-B-2021** | 19 | 9.56 | 0.36 |  | 628.58 | 41.53 |  | 560.32 | 39.41 |  | 115.17 | 6.96 |  | 40.48 | 3.26 |
| 5-B-2022 | 8 | 9.11 | 0.91 |  | 572.00 | 28.69 |  | 531.63 | 31.78 |  | 107.94 | 5.20 |  | 39.56 | 3.91 |
| 5-B-2023 | 5 | 10.40 | 0.74 |  | 604.80 | 15.02 |  | 546.40 | 11.65 |  | 110.80 | 2.86 |  | 36.48 | 2.34 |
| 5-B-2024 | 2 | 9.65 | 0.07 |  | 602.50 | 2.12 |  | 538.00 | 8.49 |  | 120.50 | 0.42 |  | 39.00 | 0.14 |
| 5-C-2018 | 7 | 9.89 | 0.34 |  | 726.00 | 29.92 |  | 648.14 | 24.84 |  | 152.29 | 6.27 |  | 46.01 | 2.94 |
| **5-C-2019** | 73 | 10.62 | 0.46 |  | 638.52 | 43.38 |  | 562.00 | 44.49 |  | 139.75 | 10.07 |  | 37.58 | 2.11 |
| **5-C-2020** | 364 | 10.71 | 0.41 |  | 664.38 | 54.87 |  | 595.07 | 48.46 |  | 141.31 | 11.82 |  | 38.85 | 3.28 |
| **5-C-2021** | 226 | 10.65 | 0.61 |  | 640.00 | 55.60 |  | 574.72 | 53.36 |  | 123.71 | 9.97 |  | 37.61 | 3.03 |
| **5-C-2022** | 134 | 10.20 | 0.72 |  | 613.09 | 42.78 |  | 561.04 | 38.65 |  | 115.82 | 7.35 |  | 37.69 | 2.30 |
| **5-C-2023** | 71 | 9.86 | 0.96 |  | 608.62 | 59.56 |  | 564.25 | 53.70 |  | 114.72 | 10.82 |  | 38.74 | 2.02 |
| **6-B-2017** | 106 | 10.49 | 0.24 |  | 684.30 | 38.19 |  | 615.88 | 41.58 |  | 143.66 | 8.04 |  | 40.76 | 2.22 |
| **6-B-2018** | 86 | 11.35 | 0.49 |  | 677.28 | 42.11 |  | 601.92 | 44.56 |  | 141.91 | 9.40 |  | 37.40 | 2.82 |
| **6-B-2019** | 217 | 11.00 | 0.28 |  | 656.42 | 48.13 |  | 577.42 | 49.92 |  | 140.40 | 9.16 |  | 37.36 | 2.60 |
| **6-B-2020** | 129 | 10.57 | 0.29 |  | 628.14 | 54.88 |  | 562.24 | 61.20 |  | 131.41 | 12.63 |  | 37.29 | 3.19 |
| **6-B-2021** | 28 | 10.61 | 0.31 |  | 575.68 | 32.66 |  | 518.54 | 41.64 |  | 107.54 | 6.22 |  | 33.94 | 1.76 |
| **6-B-2022** | 16 | 10.30 | 0.22 |  | 602.06 | 30.94 |  | 534.31 | 36.80 |  | 109.75 | 5.73 |  | 36.53 | 1.34 |
| **6-C-2017** | 17 | 10.60 | 0.42 |  | 664.59 | 28.55 |  | 600.82 | 23.65 |  | 139.45 | 6.01 |  | 39.24 | 1.70 |
| 6-C-2018 | 4 | 10.83 | 0.22 |  | 740.50 | 22.96 |  | 665.00 | 22.42 |  | 155.50 | 4.80 |  | 42.78 | 1.69 |
